# Dual Lesions: A Diagnostic Dilemma

**DOI:** 10.1155/2013/539234

**Published:** 2013-07-18

**Authors:** M. P. V. Prabhat, Prasannasrinivas Deshpande, Sarat Gummadapu, Suresh Babburi, Raja Lakshmi Chintamaneni, Bhavana Sujanamulk

**Affiliations:** ^1^Department of Oral Medicine and Radiology, Dr. Sudha and Nageswara Rao Siddhartha Institute of Dental Sciences, Chinnaoutpalli, Krishna District, Gannavaram, Andhra Pradesh 521286, India; ^2^Department of Oral Pathology, Dr. Sudha and Nageswara Rao Siddhartha Institute of Dental Sciences, Chinnaoutpalli, Krishna District, Gannavaram, Andhra Pradesh 521286, India

## Abstract

Glandular odontogenic cyst (GOC) is a rare aggressive developmental cyst of the jaw. It most commonly occurs in middle-aged people with mandible anterior region being the most affected site. This lesion can present as a unilocular or multilocular radiolucency and has high recurrence rate. The histopathologic features of the GOC are complex and often coincide with the features of dentigerous cyst, radicular cyst, and low-grade central mucoepidermoid carcinoma (CMEC). At times, the microscopic features are so similar to central low-grade mucoepidermoid carcinoma that it becomes highly impossible to distinguish the two entities even with various advanced investigations. The reported case represents one such diagnostic dilemma occurring in the maxilla which is a rare site, and the lesion/s appeared as two distinct entities, that is, GOC and CMEC on either aspects of the same side of maxilla clinically, yet showing continuity on advanced imaging and demonstrating histopathological perplexity.

## 1. Introduction 

In 1987, Padayachee and Van Wyk [1] gave the first description of this kind of cyst and later the term “Glandular Odontogenic Cyst” was introduced in 1988 by Gardner et al. [2]. It is also known as “mucoepidermoid cyst” and “sialo-odontogenic cyst.” Clinically it has a slight predilection for men and occurs mostly in middle-aged persons. Most of the reported cases have occurred in the anterior mandible. Radiographically, they are usually multilocular cystic lesions, although unilocular lesions have been reported. They are aggressive lesions and often reach large dimensions. Nevertheless, none of the clinical or radiographic features of GOC are pathognomonic [3].

The histologic features of GOC have been described in detail by several authors [1, 2, 4, 5]. Various histologic features overlap with other entities such as dentigerous cyst, mucous metaplasia in odontogenic cyst, radicular cyst, low-grade central mucoepidermoid carcinoma, and botryoid cyst. The number of typical features necessary for the diagnosis of GOC remains unclear, and there are no specific stains that distinguish GOC from similar lesions [4, 5]. 

Central mucoepidermoid carcinoma is an extremely rare tumor, representing about 2 to 4% of all mucoepidermoid carcinomas. They are histologically low-grade cancers, usually affecting the mandible as uni- or multilocular radiographic lesions [6]. 

An article with extensive study on histopathologic features and variants of GOC states that GOC and CMEC may possibly be related, but definitive evidence is still awaited [5]. 

## 2. Case Report

**Figure 1:**
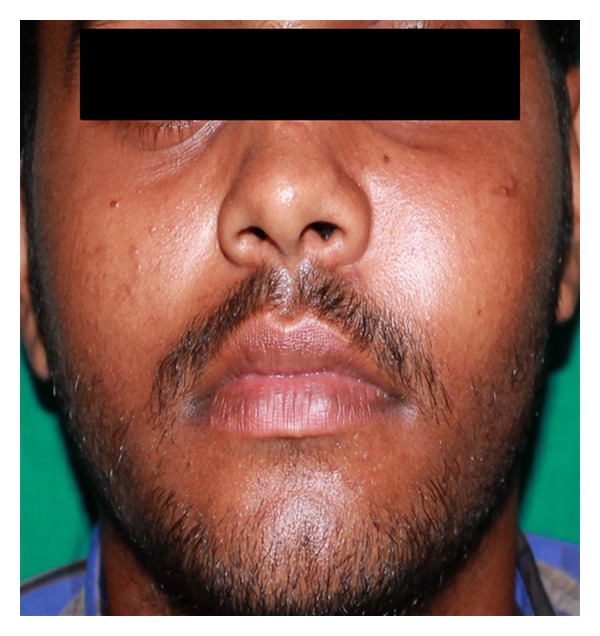
Profile picture showing swelling over the left middle third of face.

**Figure 2:**
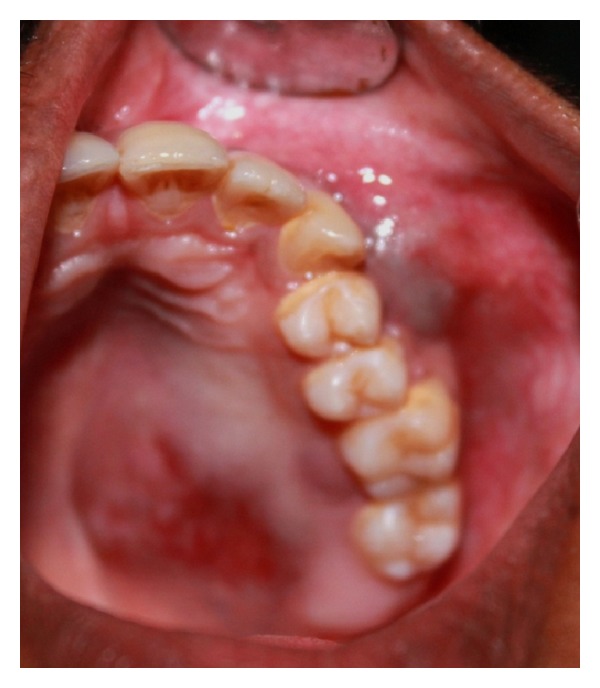
Intraoral swelling over left maxilla.

A 19-year-old young male reported with swelling in the left side of face since six months. It was insidious in onset with no history of preceding tooth ache or trauma. Swelling was gradually increasing to attain the present size and was associated with mild occasional pain. There were no symptoms of paraesthesia, diplopia, and sinus involvement. His past medical, dental, and personal histories were noncontributory. Extra-oral swelling on left mid-third region measuring about 5 × 4 cms was noted (Figure 1). The overlying surface appeared stretched with no secondary changes. Intraorally, swelling had obliterated the buccal vestibule from 23 to 25 regions which had a bluish hue and was nontender, soft, and fluctuant with areas of decortications. Palatally a well-defined swelling was seen on left posterior hard palate approximately of 3 × 3.5 cms. Surface had areas of erythema and ulceration. On palpation it was mildly tender due to superficial ulceration and firm in consistency. Grade I mobility was noted with maxillary left premolars (Figure 2).

The history and clinical examination led to an impression of low-grade central salivary gland lesion, namely, mucoepidermoid carcinoma and adenoid cystic carcinoma. Differentials included other odontogenic cysts and tumors, namely, unicystic ameloblastoma, keratocystic odontogenic tumor, and glandular odontogenic cyst. 

**Figure 3:**
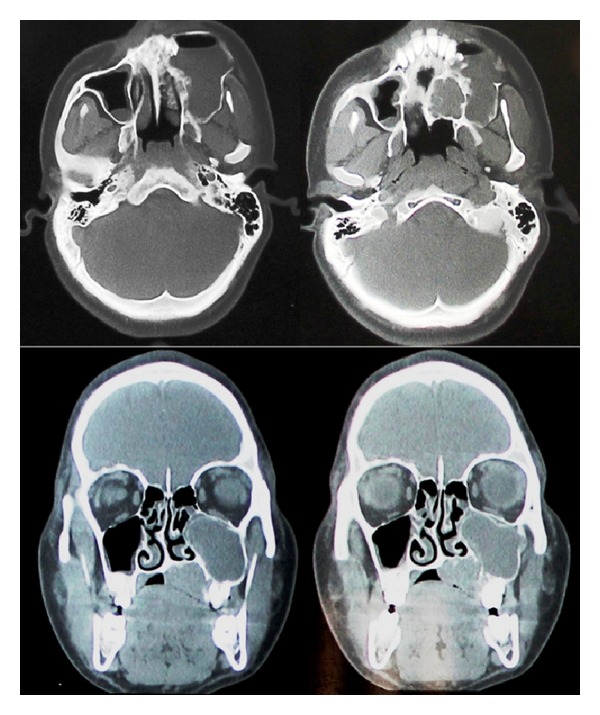
Axial and coronal CT views showing large osteolytic lesion/s involving left maxilla with continuity of buccal and palatal lesions.

 Orthopantomograph showed multilocular radiolucency over the left maxillary alveolus region as well as large diffuse radiopaque haziness involving the left maxillary sinus region extending posteriorly and causing erosion and obliteration of pterygomaxillary fissure. On CT evaluation an expansile lesion measuring 7 × 4 × 3 cms was observed on left maxilla causing expansion of buccal cortex and deviation of left lateral wall of the nose. The lesion had pushed the lower border of sinus superiorly causing near complete compression of sinus. Inferiorly destruction of the bone was noted in alveolus and palate which was multilocular in pattern. The soft tissue density was seen extending approximately 2 × 1.5 cms into the oral cavity with bone resorption whereas cortical expansion with areas of decortications was seen on buccal aspect. In anterior sections the palatal and buccal lesions appeared as two distinct entities with intact bone in between the two lesions whereas the sections of the posterior aspect of maxilla showed advanced bony destruction with continuity of the buccal and palatal lesions (Figure 3).

Aspiration from buccal lesion yielded brownish color viscous fluid and blood from palatal lesion. 

**Figure 4:**
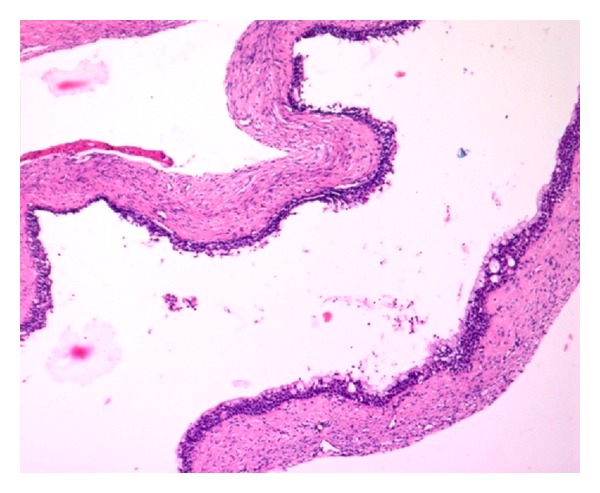
Cystic lining with connective tissue stroma (H&E 20x).

**Figure 5:**
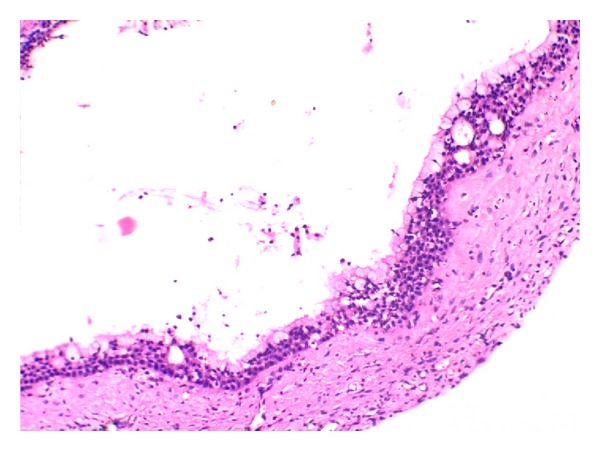
Pseudostratified squamous epithelium with multiple microcysts (H&E 20x).

**Figure 6:**
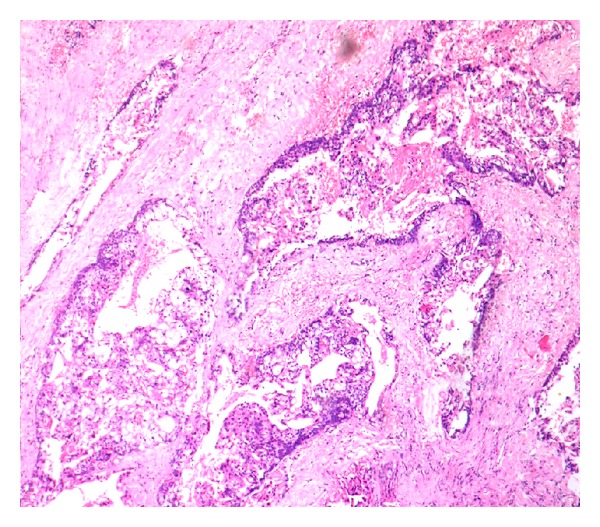
Islands of epithelium comprising mucous cells, epidermoid cells, and intermediate cells (H&E 20x).

**Figure 7:**
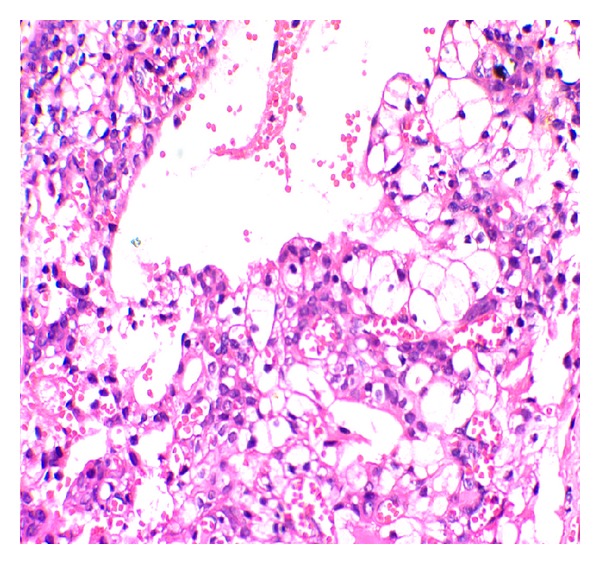
Mucous cells, epidermoid cells, and clear cells (H&E 40x).

Similarly biopsy was performed individually at both sites. Haematoxylin and eosin staining of buccal lesion showed cystic lining with ciliated pseudostratified squamous epithelium. Goblet cells with multiple microcysts were also noted. The connective tissue stroma was seen with mild inflammatory cells, all of which lead to a diagnosis of glandular odontogenic cyst (Figures 4 and 5). The lesion over the palate showed no evidence of cystic lining but, in contrast, showed presence of infiltrating islands of epithelium comprising mucous cells, epidermoid cells, and intermediate cells. Clear cells in some islands whereas cystic areas in some islands were noted with foci of inflammatory cell infiltrates in the connective tissue leading to a diagnosis of mucoepidermoid carcinoma (Figures 6 and 7).

**Figure 8:**
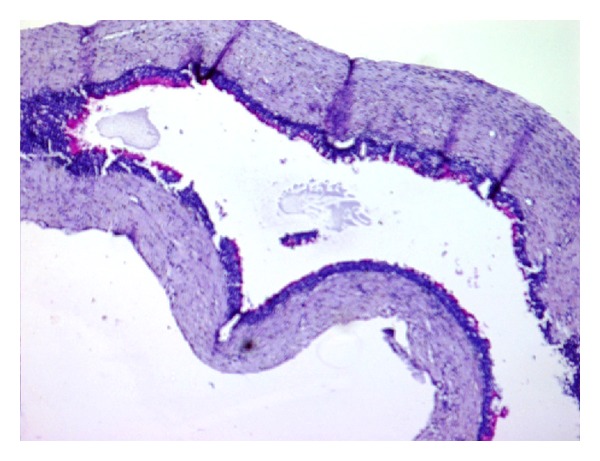
Photomicrograph of the lesion showing glandular cystic lining with mucicarmine positive mucus cells (H&E, 10x).

**Figure 9:**
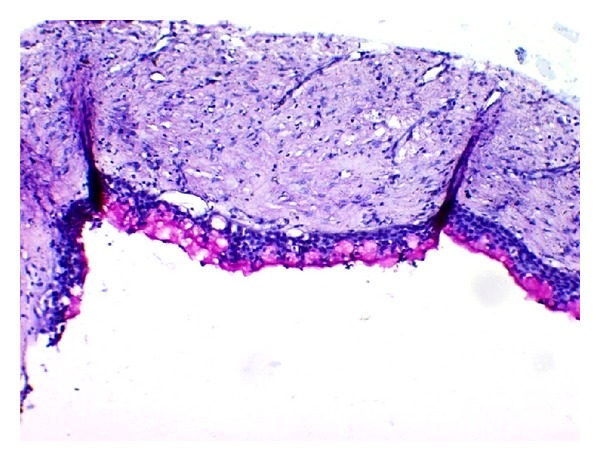
High power photomicrograph of the lesion showing glandular cystic lining with mucicarmine positive mucus cells (H&E, 20x).

**Figure 10:**
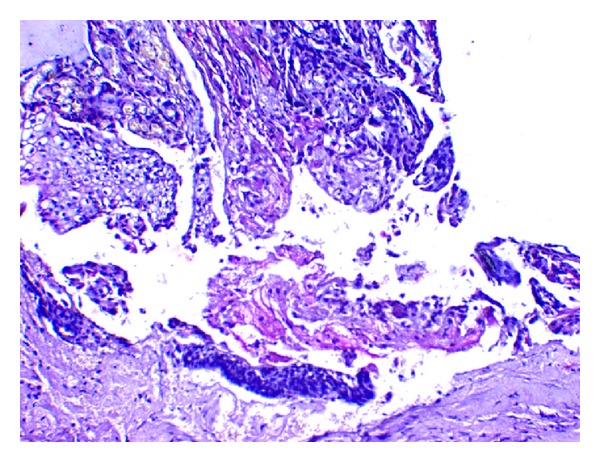
Photomicrograph of the lesion showing mucus cells (mucicarmine positive), clear cells, and intermediate cells (H&E, 10x).

**Figure 11:**
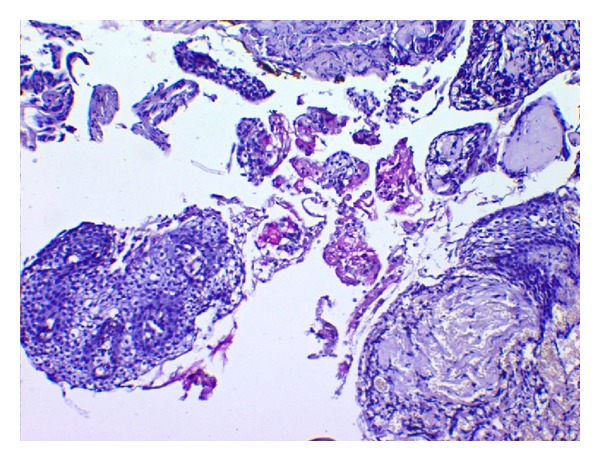
Photomicrograph of the lesion showing mucus cells (mucicarmine positive), clear cells, and intermediate cells (H&E, 10x).

No clear transition zone of carcinomatous change was noticeable in all the deeper fields examined on excisional biopsy. Mucicarmine staining showed glandular cystic lining with mucicarmine positive mucus cells (Figures 8 and 9) and palatal lesion showing mucicarmine positive mucus cells, clear cells, and intermediate cells with mild mucicarmine positive mucous pooling (Figures 10 and 11).

The patient rejected the further investigations which were advised due to monetary constraints. 

A conservative approach in the management was followed considering the age of the patient. Complete enucleation with extensive curettage was carried out, and patient was recalled every 3 months. No signs of recurrence are seen after eight months of follow-up.

## 3. Discussion 

The clinical features in the present case report present a diagnostic dilemma whether to consider it as a single lesion which presents with various clinical features or as two distinct lesions coexisting on the same side of maxilla. Mucoepidermoid carcinoma arising in maxilla was considered as it presents with solid and cystic variant. However a cyst on buccal aspect with a tumor on palate or a sinonasal pathology was also suspected. 

 Primary central MEC has been reported in the first to seventh decades; however, cases occurring in the fourth and fifth decades are most common. It has slight predilection for posterior mandible and often seen in females. MEC usually presents as a painless swelling. Pain, paraesthesia, numbness, and tooth mobility are usually occasional and late findings. Radiographically they may appear as unilocular or multilocular with or without cortical plate disruption [7]. Based on their clinical behavior they are classified as high- and low-grade MEC, and the present case was a low grade variant.

GOCs are relatively uncommon cysts first reported in 1987. Ever since then, they have remained as interesting controversial favorites for the researchers all over the world. The lesion was initially referred to as a “sialo-odontogenic cyst” and believed to have salivary gland origin, but due to lack of evidence the term “glandular odontogenic cyst” was later adopted by the World Health Organization in 1992 [8].

In the near past, numerous case reports and short series have been reported on GOCs. Therefore, the GOC, although rare, is now a relatively well-known entity. Nevertheless there are no definitive or pathognomonic clinical, radiographic, or histopathological features which aid in diagnosis.

In our case on correlating all the clinical, radiographic, and histopathological findings there were bizarre and overlapping features of GOC and CMEC. Clinically, on buccal aspect, presence of cystic swelling and blood on aspiration was noted in contrast to firm swelling and brownish color viscous fluid on palatal aspect. Radiographic findings revealed buccal cortical expansion with areas of decortications suggestive of cystic lesion whereas palatal lesion showed bony resorption with soft tissue infiltrate supporting the clinical findings of tumor. However detailed radiographic evaluation showed continuity in both aspects further enhancing the diagnostic predicament.

A well-known fact of GOC is that it mimics various pathologies occurring in the jaws. In our case, the presence of cystic lining with ciliated pseudostratified squamous epithelium, goblet cells, multiple microcysts, and mucicarmine positive mucous cells in the buccal lesion suggested GOC while palatal lesion showed infiltrating islands of epithelium comprising mucous cells, epidermoid cells and intermediate cells along with clear cells, mucicarmine positive mucus cells with mucous pooling providing a diagnosis of MEC. Overlapping feature on deeper field examinations with no clear field of transition further complicated the diagnosis. A recent article in 2011 analysing 46 cases of GOC with special emphasis on microscopic criteria for diagnosis concludes that at this point of time no enough information is available to determine whether GOC and CMEC share a histopathological spectrum or whether MEC-like changes in GOCs are associated with malignant behaviour [5].

Immunohistochemistry may aid to a smaller extent in differentiating GOC and CMEC. Assessment of the cytokeratin (CK) profile of central MEC and GOC overlaps to a greater extent, but expression of CKs 18 and 19 could be useful in their differential diagnosis [9]. 

## 4. Conclusion

Oral cavity may rarely house controversial coexisting lesions which may challenge the diagnosis and acumen of clinician. A specialized oral physician should be aware of such pathologies and work towards solving the mystery as the exact diagnosis is necessary to render proper management. The present case highlights one such diagnostic dilemma which was attempted to be solved with the available resources.
